# Hemoglobin concentration does not impact 3-month outcome following acute ischemic stroke

**DOI:** 10.1186/s12883-018-1082-8

**Published:** 2018-06-02

**Authors:** Kartavya Sharma, Daniel J. Johnson, Brenda Johnson, Steven M. Frank, Robert D. Stevens

**Affiliations:** 1 Department of Anesthesiology and Critical Care Medicine, Johns Hopkins University School of Medicine, Baltimore, USA; 2 Department of Neurology, Johns Hopkins University School of Medicine, Baltimore, USA; 3 Department of Neurology, University of Kansas Medical Center, Mailstop 2012, 3901 Rainbow Blvd, Kansas City, KS 66160 USA; 4 Department of Neurosurgery, Johns Hopkins University School of Medicine, Baltimore, USA

**Keywords:** Ischemic stroke, Anemia, Hemoglobin, Transfusion, Disability, Mortality, Charlson comorbidity index

## Abstract

**Background:**

There is uncertainty regarding the effect of anemia and red blood cell transfusion on functional outcome following acute ischemic stroke. We studied the relationship of hemoglobin parameters and red cell transfusion with post stroke functional outcome after adjustment for neurological severity and medical comorbidities.

**Methods:**

Retrospective cohort study of 536 patients discharged with a diagnosis of ischemic stroke from a tertiary care hospital between January 2012 and April 2015. Hemoglobin level at hospital admission, lowest recorded value during hospitalization (nadir), delta hemoglobin (admission minus nadir), red cell transfusion during hospitalization were noted. Charlson Comorbidity Index (CCI) was computed as a summary measure of medical comorbidities. A multivariable logistic regression model was used to determine risk-adjusted odds of unfavorable outcome, defined as a modified Rankin Score of > 2.

**Results:**

Anemia was present on hospital admission in 31% of patients. Forty five percent of patients had unfavorable outcome. In the univariable analysis increasing age, admission National Institutes of Health Stroke Scale (NIHSS), CCI, nadir hemoglobin, delta hemoglobin and blood transfusion were associated with unfavorable outcome. In the multivariable model, only increasing age, CCI and NIHSS remained associated with unfavorable outcome. No quadratic association was found on repeating the model to identify a possible U-shaped relationship of hemoglobin with outcome.

**Conclusions:**

Our findings contradict prior observational studies and highlight an area of clinical equipoise regarding the optimal management of anemia in patients hospitalized for ischemic stroke. This uncertainty could be addressed with appropriately designed clinical trials.

## Background

Anemia is a frequent comorbid or complicating factor in patients with ischemic stroke, yet the influence of hemoglobin (Hb) concentration on stroke outcome is a matter of considerable uncertainty. Increases in mortality following ischemic stroke have been associated with abnormally low Hb level [1–5] as well as abnormally high Hb level [6], or both high and low levels in the same cohort [7]. Observational studies have suggested an unfavorable impact of admission anemia on long term post-stroke functional outcome [3, 7]. However, one study found this association only in a subgroup of patients with less severe strokes [8] and another large cohort study with meta-analysis did not find any association with functional outcome [2].

Several factors may contribute to these differences in findings. First, Hb-related exposure/predictor variables are not consistently reported across studies. For example, dynamic assessments of anemia after ischemic stroke, such as the decrease in Hb or the nadir Hb, were shown to be independently predictive of worse outcome in one report [9], however most studies have not evaluated anemia in this way. Second, statistical models vary widely across studies particularly regarding the use of multivariable approaches, and in the selection of covariates used in the multivariable models. For example, it is plausible that red blood cell (RBC) transfusion will modify any presumed impact of anemia on stroke pathophysiology, yet RBC transfusion is not consistently adjusted for across studies. Lastly, anemia can be regarded as a marker of underlying medical illnesses which can influence outcome independently of any direct mechanistic effect on the ischemic brain. Variability in study results may therefore reflect unmeasured or unreported confounders (e.g. acquired immune deficiency syndrome, malignancy, malnutrition and liver failure) which can influence Hb level, functional outcome or both.

To clarify these effects, we examined the relationship of Hb indices including admission, nadir, discharge and change in Hb level with 3-month functional outcome in a large hospital-based stroke registry, adjusting for RBC transfusion and for medical comorbidities using the comprehensive Charlson Comorbidity Index (CCI).

## Methods

We queried the Johns Hopkins Hospital stroke registry, a prospective database, for patients aged 18 and above who were discharged with a diagnosis of ischemic stroke between Jan 1st 2012 and Apr 30th 2015. For patients admitted more than once with acute ischemic stroke, we considered only the earliest hospitalization. Out of a total of 802 patients, modified Rankin Scale (mRS) at 3 months was available for 536 patients. These patients were cross-referenced against a separate Blood Product Utilization Database established by one of the co-investigators (SMF). Predictor variables were clinical characteristics including pre-admission CCI, laboratory values and record of blood product transfusions during the hospitalization. Anemia was defined as per the World Health Organization as a serum Hb of < 13 g/dL in men and < 12 g/dL in women [10]. The principal outcome variable was unfavorable functional status defined as mRS > 2 at 3 months follow up.

Statistical analyses: Exploratory univariable analysis was performed to determine strength of the association between potential predictors and the principal outcome variable. Nominal logistic regression was used for continuous variables and Pearson’s chi-squared test for categorical variables. Covariates having a *P* value < 0.1 on univariable analysis were used in a multiple logistic regression model with unfavorable outcome as the dependent variable. Of note, despite having no association on univariable testing (*P* = 0.59), intravenous tissue plasminogen activator (tPA) administration was included in the multivariable models because of its well-established efficacy in improving stroke outcomes. We tested using separate models, the following Hb indices as continuous variables for strength of association with outcome: admission, nadir, last recorded serum Hb concentration, as well as delta (difference between admission and nadir) Hb. In secondary analyses, we repeated our model to address two issues. First, to investigate a possible quadratic relationship of Hb with outcome, we included Hb squared as a continuous variable. Second, instead of linear or quadratic Hb values, we used anemia as a categorical predictor (Hb < 12 g/dl for women and < 13 g/dl for men) [10]. *P* value threshold was set at ≤0.05 for statistical significance. Analyses were performed using JMP®, Version 12.1 (SAS Institute Inc., Cary, NC).

## Results

A total of 536 patients were included in the study. Patient characteristics and hemoglobin parameters are provided in Table 1. The prevalence of anemia on admission was 31% in the study sample. The proportion of anemic women (38%) was significantly higher than men (26%), *P* = 0.002. During the hospitalization, the prevalence of anemia increased to 59% based on nadir Hb values.

**Table 1:** Patient characteristics (*N* = 536)

Characteristic	No. (%) of Patients
Female	244 (45.5)
Age, mean (SD), y	62 (15)
Admission NIHSS score	
Median (IQR)	3 (5)
Mean (SD)	5.2 (5.5)
NIHSS < 10	450 (84)
Hemoglobin parameters	
Admission Hb, mean (SD), g/dl	13.2 (1.9)
Nadir Hb, mean (SD), g/dl	11.7 (2.3)
Delta Hb, median (IQR), g/dl	1.1 (1.9)
Charlson Comorbidity Index, median (IQR)	3 (3)
Body mass index ≥30	58 (11)
Congestive heart failure	66 (12)
Hypertension	90 (17)
Diabetes mellitus	157 (29)
Renal disease	63 (12)
Liver disease	14 (3)
HIV positive	6 (1)
Intravenous alteplase administered	39 (7)
Length of stay, median (IQR)	3 (5)
Red blood cell transfusion	28 (5)
3-month modified Rankin scale score	
0	98 (18)
1	90 (17)
2	105 (20)
3	121 (23)
4	68 (13)
5	20 (4)
6	34 (6)

Abbreviations: *HIV* human immunodeficiency virus, *IQR* interquartile range, *NIHSS* National Institutes of Health stroke scale, *SD* standard deviation

Three-month unfavorable outcome, defined as a mRS > 2, was noted in 243 (45%) patients, of whom 34 (6%) died within the 3-month follow up period. In the univariable analysis, greater age, higher NIHSS score, CCI, lower nadir Hb, larger delta Hb, and RBC transfusion were associated with unfavorable outcome (Table 2). In the multivariable model, age, CCI and NIHSS remained associated with worse outcome (Table 2). However, the associations of nadir or delta Hb and RBC transfusion with unfavorable outcome were not significant in the risk-adjusted analysis (Table 3). Modeling performed with admission Hb as a dichotomized value (anemia vs no anemia) did not reveal significant association on multivariable analysis (Table 3). In post hoc analyses using only death as the outcome of interest, Hb indices were again not associated with mortality. There was no quadratic relationship of Hb with mortality or functional outcome (models not shown).

**Table 2:** Univariable and multivariable predictors of unfavorable outcome (mRS > 2)

	Univariable Analysis^a^		Multivariable analysis^b^	
	OR (95% CI)	P Value	Adjusted OR (95% CI)	P value
Age	1.02 (1.01–1.04)	< 0.001	1.02 (1.01–1.04)	0.001
NIHSS score	1.26 (1.19–1.33)	< 0.001	1.30 (1.23–1.39)	< 0.001
IV alteplase use	0.84 (0.42–1.61)	0.59	0.19 (0.07–0.46)	< 0.001
Charlson Comorbidity Index	1.30 (1.17–1.44)	< 0.001	1.25 (1.11–1.41)	< 0.001
Nadir Hb	0.82 (0.76–0.89)	< 0.001	0.99 (0.89–1.10)	0.84
Delta Hb	1.40 (1.20–1.60)	< 0.001		
Anemia during hospitalization	1.60 (1.15–2.33)	0.006		
Red blood cell transfusion	2.67 (1.21–6.31)	0.013	1.45 (0.49–4.40)	0.50

Abbreviations: *CI* Confidence interval, *OR* Odds ratio, *Hb* Hemoglobin

^a^Odds ratios are per unit change for continuous variables (Age,y; NIHSS score; Charlson Comorbidity Index; Nadir Hb, g/dl; Delta Hb, g/dl; Body Mass Index)

^b^Only one multivariable model shown here. Results for models with other Hb indices shown in Table 3.

**Table 3:** Unadjusted and adjusted odds ratios for unfavorable outcome with different hemoglobin variables

	OR (95% CI)	P value	Adjusted OR^a^ (95% CI)	P value
Admission Hb	0.96 (0.88–1.05)	0.47	1.05 (0.94–1.17)	0.34
Nadir Hb	0.82 (0.76–0.89)	< 0.001	0.99 (0.89–1.1)	0.84
Last Hb	0.82 (0.76–0.89)	< 0.001	0.98 (0.88–1.09)	0.75
Delta Hb	1.40 (1.20–1.60)	< 0.001	1.14 (0.97–1.34)	0.09
Nadir Hb in patients with NIHSS < 10	0.86 (0.79–0.94)	0.002	0.99 (0.88–1.1)	0.93
Anemia during hospitalization	1.60 (1.15–2.33)	0.006	0.83 (0.5–1.3)	0.41

Abbreviations: *Hb* hemoglobin

^a^Models were adjusted for age, NIHSS, Charlson Comorbidity Index, delta Hb and red blood cell transfusion

## Discussion

In this large cohort of patients admitted to a tertiary care center with ischemic stroke, anemia was present on admission in nearly one third of cases. Moreover, anemia developed after admission in 28% of patients who were initially hospitalized without anemia. Nevertheless, in carefully risk adjusted models, neither anemia on admission nor anemia during hospitalization, nor any Hb-associated variable, nor RBC transfusion was significantly associated with functional status at 3 months. Taken in the context of other published studies, the results illustrate an area of significant uncertainty in the evaluation and management of patients with ischemic stroke.

Putative mechanisms for how anemia may influence the pathophysiology and outcome of ischemic stroke include reduction of oxygen carrying capacity to the penumbral regions [11], the generation of a hyperkinetic thrombogenic state especially in acute blood loss [12] and the association with a proinflammatory state [13]. However, findings from studies looking at the association of Hb with functional outcome after stroke have been inconsistent. Tanne et al. reported significantly increased odds of combined death and disability (Barthel Index < 75) at 1 year in patients with anemia [7]. Similarly, Milionis et al. reported significantly increased odds of poor functional status measured with mRS at 3 months and 1 year in anemic vs non-anemic patients [3]. In contrast, Hao et al. found no association between anemia and measures of disability in their cohort and meta-analysis of similar studies published in the period 2007–2013 [2].

Increased mortality in anemic patients has been demonstrated in studies with follow up periods ranging from 1 month to 3 years [1–5]. This seemingly intuitive relationship has not borne out uniformly. Furlan et al. noted no association of low Hb with 7-day and 30-day mortality but slight increase in 90-day mortality [6]. Some caveats bear mentioning when interpreting these data. Two of these large cohort studies did not incorporate stroke severity as a covariate influencing mortality [4, 5]. There also seems to be publication bias favoring reports with increased odds of mortality in anemic patients with stroke [5, 14].

Some data suggest that both low and high Hb may be linked with increased mortality. In the study by Furlan et al., abnormally high Hb was robustly associated with increased mortality at all follow up intervals [6]. Tanne et al. found increased mortality rates at both low and high Hb concentrations [7]. Thrombogenic effects and compromised collateral flow have been postulated as mechanisms worsening outcomes in patients with supranormal serum Hb values. An alternative explanation would be that both extremes of Hb concentration are biomarkers of systemic medical comorbidities which are the true drivers of mortality. Indeed, we did not find any such quadratic association of mortality with Hb parameters.

It has been also been postulated that with increasing severity of stroke, the relative impact of anemia on outcome may become insignificant due to the extent of neurological injury [8, 9, 15]. Sico et al. reported an association of low hematocrit (< 30%) with combined outcome of death and discharge to hospice in patients with mild to moderate stroke (NIHSS < 10) but not in those with more severe strokes [8]. In another set of studies, Kellert et al. implicated post-admission drop in Hb levels in worsening functional outcome in thrombolysed patients admitted to the stroke unit, but found no such association in their neurological intensive care unit patients with more severe illness [9, 15]. In our cohort, however, there was no association of Hb parameters with mortality or functional outcome in the subgroup of patients with NIHSS < 10 (*n* = 450, Table 3).

Strengths of this study include the relatively large sample size, adjustment for stroke severity and the use of the CCI as a summary measure for comorbidity information. Due to the large number of conditions that may be covariant with anemia and mortality, a summary measure provides the convenience of a single number that adequately captures information from individual comorbidities [16]. The CCI has been in extensive use in administrative databases and has been validated as a prognostic indicator in ischemic stroke as well [17, 18]. Consistent use of such a measure will be useful in comparing and pooling data across studies in future. Limitations of the study include the retrospective design and potential selection bias due to exclusion of patients with missing mRS data. We did not have documented pre-stroke mRS for the study patients. Change in mRS would certainly be a more accurate outcome measure than post-stroke value alone. Lastly, we could not extract information about mechanism of stroke in our patients. It is entirely plausible that different stroke subtypes may be differentially affected by abnormal hemoglobin levels.

In summary, in our cohort of patients with ischemic stroke, admission, nadir or change in Hb were not predictive of 3-month functional outcome. It is possible that mild to moderate anemia, other than being a marker of illness, does not materially affect prognosis from ischemic stroke. Perhaps the central nervous system adaptation to chronic anemia may be protective in the setting of acute ischemia, similar to the neuroprotective effect of hypoxic preconditioning in a rodent model of ischemic stroke [19]. Another potentially important factor is the intrinsic biological heterogeneity in populations of patients with ischemic stroke [20]. An emerging body of research is demonstrating that variance in the risk, presentation and outcomes of ischemic stroke is determined to a significant degree by underlying genetic factors [21], suggesting that anemia and RBC transfusion could have differential effects depending on underlying (and yet insufficiently characterized) patient-specific characteristics.

Inconsistent results from available studies suggest a state of clinical equipoise regarding the optimal management of anemia in patients with acute ischemic stroke. Resolving this uncertainty could involve 3 linked strategies. First, prospective studies are needed with sample sizes adequately powered to answer primary hypotheses. Such studies must use carefully designed modeling approaches that control for a range of potential confounders and focus on functional outcomes in addition to mortality. Consistency in the use of comorbidity summary measures and outcome measures may enable pooling of data across studies to overcome the limitations of sample size. Second, research is needed to discover and validate sensitive and specific biomarkers to guide management of anemia (e.g. timing of transfusion, optimal Hb cutoffs) in stratified subsets or individuals with ischemic stroke. Third, carefully designed and adequately powered randomized controlled trials (preferably biomarker-guided) are warranted to address primary questions on the role of higher versus lower Hb management thresholds in ischemic stroke populations.

## Conclusion

Our study demonstrated no association of hemoglobin parameters with mortality and 3-month functional outcome, which contradicts prior observational evidence. This uncertainty could be addressed with appropriately designed clinical trials.

## Abbreviations

CCI: Charlson Comorbidity Index
CI: Confidence interval
Hb: Hemoglobin
HIV: Human immunodeficiency virus
IQR: Interquartile range
mRS: Modified rankin scale
NIHSS: National Institutes of Health Stroke Scale
OR: Odds ratio
RBC: Red blood cell
SD: Standard deviation
tPA: Tissue plasminogen activator

## References

[1] Nybo M, Kristensen SR, Mickley H, Jensen JK (2007). The influence of anaemia on stroke prognosis and its relation to N-terminal pro-brain natriuretic peptide. Eur J Neurol.

[2] Hao Z, Wu B, Wang D, Lin S, Tao W, Liu M (2013). A cohort study of patients with anemia on admission and fatality after acute ischemic stroke. J Clin Neurosci.

[3] Milionis H, Papavasileiou V, Eskandari A, D’Ambrogio-Remillard S, Ntaios G, Michel P (2015). Anemia on admission predicts short- and long-term outcomes in patients with acute ischemic stroke. Int J Stroke.

[4] Huang WY, Chen IC, Meng L, Weng WC, Peng TI (2009). The influence of anemia on clinical presentation and outcome of patients with first-ever atherosclerosis-related ischemic stroke. J Clin Neurosci..

[5] Barlas RS, Honney K, Loke YK, McCall SJ, Bettencourt-Silva JH, Clark AB, et al. Impact of hemoglobin levels and Anemia on mortality in acute stroke: analysis of UK regional registry data, systematic review, and meta-analysis. J Am Heart Assoc. 2016;5 10.1161/jaha.115.003019. 10.1161/JAHA.115.003019 PMC5015269 27534421

[6] Furlan JC, Fang J, Silver FL (2016). Acute ischemic stroke and abnormal blood hemoglobin concentration. Acta Neurol Scand.

[7] Tanne D, Molshatzki N, Merzeliak O, Tsabari R, Toashi M, Schwammenthal Y (2010). Anemia status, hemoglobin concentration and outcome after acute stroke: a cohort study. BMC Neurol.

[8] Sico JJ, Concato J, Wells CK, Lo AC, Nadeau SE, Williams LS (2013). Anemia is associated with poor outcomes in patients with less severe ischemic stroke. J Stroke Cerebrovasc Dis.

[9] Kellert L, Martin E, Sykora M, Bauer H, Gussmann P, Diedler J (2011). Cerebral oxygen transport failure?: decreasing hemoglobin and hematocrit levels after ischemic stroke predict poor outcome and mortality: STroke: RelevAnt impact of hemoGlobin, hematocrit and transfusion (STRAIGHT)--an observational study. Stroke.

[10] Fagnoul D, Combes A, De Backer D (2014). Extracorporeal cardiopulmonary resuscitation. Curr Opin Crit Care.

[11] Dexter F, Hindman BJ (1997). Effect of haemoglobin concentration on brain oxygenation in focal stroke: a mathematical modelling study. Br J Anaesth.

[12] Kim JS, Kang SY (2000). Bleeding and subsequent anemia: a precipitant for cerebral infarction. Eur Neurol.

[13] Ferrucci L, Guralnik JM, Woodman RC, Bandinelli S, Lauretani F, Corsi AM (2005). Proinflammatory state and circulating erythropoietin in persons with and without anemia. Am J Med.

[14] Li Z, Zhou T, Li Y, Chen P, Chen L (2016). Anemia increases the mortality risk in patients with stroke: a meta-analysis of cohort studies. Sci Rep.

[15] Kellert L, Herweh C, Sykora M, Gussmann P, Martin E, Ringleb PA (2012). Loss of penumbra by impaired oxygen supply? Decreasing hemoglobin levels predict infarct growth after acute ischemic stroke: stroke: Relevant impact of hemoglobin, hematocrit and transfusion (STRAIGHT) - an observational study. Cerebrovasc Dis Extra.

[16] Austin SR, Wong Y-N, Uzzo RG, Beck JR, Egleston BL (2015). Why summary comorbidity measures such as the Charlson comorbidity index and Elixhauser score work. Med Care.

[17] Goldstein LB, Samsa GP, Matchar DB, Horner RD (2004). Charlson index comorbidity adjustment for ischemic stroke outcome studies. Stroke.

[18] Tessier A, Finch L, Daskalopoulou SS, Mayo NE (2008). Validation of the Charlson comorbidity index for predicting functional outcome of stroke. Arch Phys Med Rehabil.

[19] Wacker BK, Perfater JL, Gidday JM (2012). Hypoxic preconditioning induces stroke tolerance in mice via a cascading HIF, sphingosine kinase, and CCL2 signaling pathway. J Neurochem.

[20] Rostanski SK, Marshall RS (2016). Precision medicine for ischemic stroke. JAMA Neurol.

[21] Falcone GJ, Malik R, Dichgans M, Rosand J (2014). Current concepts and clinical applications of stroke genetics. Lancet Neurol.

